# Trends in Surgical Outcomes and Overall Survival Among Women Undergoing Debulking Surgery for Advanced Ovarian Cancer in the U.S: Analysis of the National Cancer Database

**DOI:** 10.3390/cancers17172884

**Published:** 2025-09-02

**Authors:** Kelly Lamiman, Michael Silver, Judy Hayek, Ryan Hanusek, Lea Sarmiento, Michael Kim, Nicole Goncalves, Ioannis Alagkiozidis

**Affiliations:** 1 Department of Gynecologic Oncology, Maimonides Medical Center, Brooklyn, NY 11220, USA; klamiman@maimo.org (K.L.); ngoncalves@maimo.org (N.G.); 2 Department of Gynecologic Oncology, SUNY Downstate Health Sciences University, Brooklyn, NY 11203, USA; lea.sarmiento@downstate.edu; 3 Department of Obstetrics and Gynecology, New York-Presbyterian Brooklyn Methodist Hospital, Brooklyn, NY 11215, USA

**Keywords:** ovarian cancer, neoadjuvant chemotherapy, primary debulking surgery, interval debulking surgery, overall survival

## Abstract

Given the rising use of neoadjuvant chemotherapy (NACT) followed by interval debulking surgery (IDS) in advanced epithelial ovarian cancer (EOC), we sought to assess practice trends in overall survival (OS), complete gross resection (R0), and postoperative mortality following debulking surgery. The National Cancer Database (NCDB) was used to identify 34,982 stage IIIC and IV EOC patients between 2010 and 2017 who underwent surgery. Annual proportions of patients receiving IDS and PDS were calculated. Median OS was estimated using the Kaplan–Meier method. Joinpoint models were fitted to evaluate surgical trends. Statistics were performed using SPSS and Joinpoint. Of 34,982 patients, 10,460 (29.9%) underwent IDS. IDS patients were older, more likely to have stage IV disease, and more likely to be non-White. Median OS was higher in the PDS group (54 vs. 38.8 months, *p* < 0.001). Postoperative 90-day mortality was lower in the PDS group (1.7% vs. 2.4%, *p* < 0.001), though IDS patients had a lower 30-day readmission rate (6.2% vs. 3.1%, *p* < 0.001). IDS patients were less likely to undergo extensive surgery (27.4% vs. 36.7%, *p* < 0.001) and more likely to achieve R0 resection (42% vs. 38.6%, *p* < 0.001). The IDS rate increased from 18.9% to 40.6% (annual percentage change (APC): 11.8%, *p* < 0.05) from 2010 to 2017. Median OS improved from 46.6 to 51 months (APC: 1.9%, *p* < 0.05), driven by the PDS cohort. The R0 resection rate rose from 34.8 to 41% (APC: 2.65%, *p* < 0.01), driven by the PDS cohort (APC: 2.83%, *p* < 0.01). Postoperative 90-day mortality decreased from 2.4% to 1.5% (APC: −4.64%, *p* < 0.05), due to a reduction in PDS patients (APC: −6.83%, *p* < 0.05). There was no change in the rate of extensive surgery over time. In conclusion, from 2010 to 2017, increased triage of patients to NACT was accompanied by a higher R0 resection rate and reduced postoperative mortality in PDS patients, with no observed detriment to OS. This data suggests improvement in case selection between IDS and PDS.

## Simple Summary

The approach to the treatment of advanced epithelial ovarian cancer has significantly changed over the past fifteen years. While surgery and chemotherapy remain the primary treatments, determining who benefits most from upfront surgery versus upfront neoadjuvant chemotherapy has been a topic of intense study in recent years. There has been rising use of neoadjuvant chemotherapy with interval debulking surgery following landmark trials showing its non-inferiority to primary debulking surgery with adjuvant chemotherapy. We sought to quantify the trend in the surgical and systemic management of advanced ovarian cancer and determine their corresponding survival and peri-operative outcomes over time. This large retrospective database study shows the real-world impact of these changes in the management of advanced epithelial ovarian cancer. The use of neoadjuvant chemotherapy and interval debulking surgery increased year over year, overall survival increased, and the 90-day mortality decreased. The primary debulking group showed improvements in the 90-day mortality and complete gross resection rate over time, suggestive of improved case selection.

## 1. Introduction

The National Cancer Institute Surveillance, Epidemiology, and End Results (SEER) database anticipates approximately 20,890 new ovarian cancer diagnoses and 12,730 deaths in 2025 [1]. The treatment for advanced epithelial ovarian cancer (EOC) is systemic platinum-based chemotherapy and surgery. Debulking surgery typically includes hysterectomy, bilateral salpingo-oophorectomy, omentectomy, lymph node dissection, and resection of all visible tumor. Surgical cytoreduction to no visible disease (R0) significantly predicts overall survival in advanced EOC [2,3,4,5,6]. If R0 is not achievable, the surgical goal becomes optimal debulking to reduce disease burden to less than 1 cm (R < 1 cm). When optimal debulking is possible, the preferred treatment sequencing is typically primary surgery followed by adjuvant chemotherapy.

In the mid-2010s, three international landmark surgical clinical trials showed non-inferior overall survival (OS) using neoadjuvant chemotherapy (NACT) with interval debulking surgery (IDS) compared to primary debulking surgery (PDS) with adjuvant chemotherapy for patients with advanced stage IIIC or IV EOC [7,8,9]. Median OS varied amongst the trials with the shortest median OS in the CHORUS trial (23 months PDS vs. 24 months IDS), compared to the Vergote et al. trial (29 months PDS vs. 30 months IDS) and SCORPION (41 months PDS vs. 43 months IDS) [7,8,9].

In addition to the established noninferior OS data, a Japanese clinical trial showed that NACT with IDS was associated with shorter operative times, fewer surgical complications, and fewer organ resections compared to PDS with adjuvant chemotherapy [10]. The authors hypothesized that the effect of NACT on reducing tumor size and ascites burden may have resulted in improved surgical tolerance and peri-operative outcomes [10]. Taken all together, the data from these four clinical trials prompted a large paradigm shift in practice patterns and the sequencing of surgery and chemotherapy in advanced EOC.

Despite the prospective randomized trials showing non-inferiority of NACT with IDS, retrospective observational studies showed OS benefit to PDS [11,12]. A recent retrospective study of the National Cancer Database (NCDB) showed that institutions that used NACT with IDS more frequently experienced a reduction in early mortality without a decrease in overall survival (OS) [13]. Trend analysis of the NCBD has also shown an increase in the use of NACT associated with decreased peri-operative mortality and increased 5-year OS [14].

Now that it has been approximately fifteen years since the publication of these landmark trials, we sought to perform a retrospective analysis of a real-world cohort to assess practice patterns and surgical trends in the United States. We designed a large epidemiological retrospective study using NCDB to assess trends in OS, complete gross resection (R0), and postoperative mortality following debulking surgery for advanced EOC given the rising use of NACT.

## 2. Materials and Methods

We identified a cohort of 34,982 patients with advanced stage III-IV EOC over the eight-year period between January 2010 and December 2017 who underwent either PDS with adjuvant chemotherapy or NACT with IDS. The NCDB is a hospital-based registry of US patients who received care at over 1500 facilities accredited by the Commission on Cancer. The database represents about 70% of newly diagnosed ovarian cancer cases in the country [15]. We excluded patients without a hysterectomy, whose primary treatment was unknown, and those without pathologically confirmed disease. The diagnostic code C56.9 (Malignant neoplasm of ovary) was used to identify cases who underwent surgery or chemotherapy within six months of diagnosis. Analysis was restricted to patients with American Joint Committee on Cancer (AJCC) Stage IIIC-IV disease and epithelial ovarian cancer histology (serous, clear cell, endometroid, mucinous) using the NCDB participant user files (PUF) dictionary ICD-O-3 codes by SEER registries. We calculated the proportion of women treated with chemotherapy first, and defined this as the NACT group. Early-stage disease, non-epithelial histology, and no treatment cases were excluded. Patients with missing data were excluded, which represented less than 3% of cases for our primary and secondary outcomes.

The primary outcome was the trend in OS over time using the Kaplan–Meier method. OS was defined as the number of months from diagnosis to death or last contact. The annual proportions of patients receiving IDS and PDS were calculated. Joinpoint models were fitted to evaluate trends in treatment type, OS, R0 resection, postoperative mortality, and extensive surgery rates. Extensive surgery was defined as procedures beyond hysterectomy, salpingo-oophorectomy, omentectomy, pelvic and para-aortic lymphadenectomy. Specifically, the NCDB data dictionary codes used to classify extensive surgery are those that contained any bowel resection (code 61/63), urinary tract resection (code 62/63), or exenteration (code 70/71/72/73/74) procedures. Statistics were performed using SPSS Version 29.0 (IBM Corp, Armonk, NY, USA) and Joinpoint with a two-sided *p*-value of <0.05 considered significant. The research was Institutional Review Board exempt as determined by the Maimonides Medical Center due to the de-identified nature of the database.

## 3. Results

### 3.1. Patient and Disease Characteristics

A total of 34,982 patients met the inclusion criteria (Table 1). Of those, 10,460 (29.9%) underwent NACT with IDS and 24,522 (70.1%) underwent PDS with adjuvant chemotherapy. In univariate analysis, patients who underwent IDS were older (65 vs. 61 years, *p* < 0.001), more likely to have stage IV disease (51.3% vs. 25.4%, *p* < 0.001), more likely to be publicly insured (55.1% vs. 44.5%, *p* < 0.001), and less likely to be White (85.6% vs. 86.9%, *p* = 0.015) compared to the PDS cohort. These findings are similar to other NCDB reported data [14].

### 3.2. Survival Analysis and Trends

**Figure 1:**
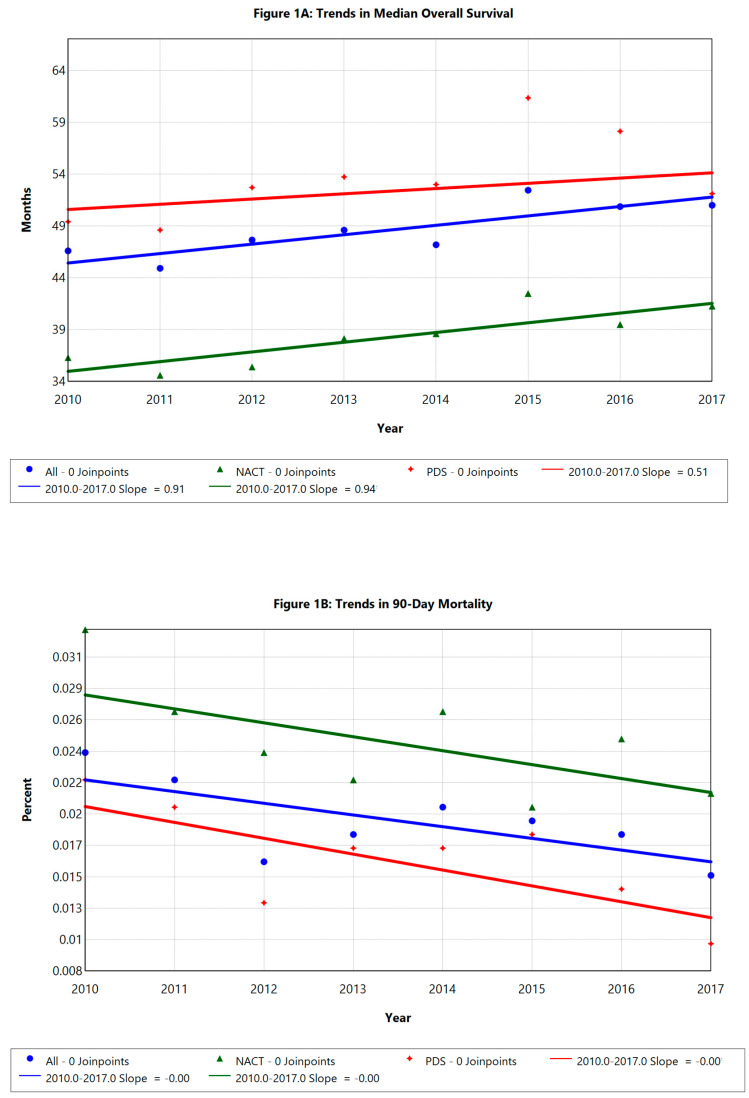
(**A**) Overall survival trend; (**B**) 90-day mortality trend. APC = annual percentage change; NACT = neo-adjuvant chemotherapy with interval debulking surgery; IDS = interval debulking surgery; PDS = primary debulking surgery.

Median OS was significantly longer in the PDS group (54 vs. 38.8 months, *p* < 0.001, Supplementary Figure S1). The median OS for all patients improved from 46.6 to 51 months over the study period from 2010 to 2017 (annual change: 1.9%, *p* < 0.05, Figure 1A). Postoperative 90-day mortality was higher in the IDS group (2.4% vs. 1.7%, *p* < 0.001, Figure 1B). Postoperative 90-day mortality decreased from 2.4% to 1.5% (annual change: −4.64%, *p* < 0.05, Figure 1B), primarily due to a reduction in mortality among PDS patients (annual change: −6.83%, *p* < 0.05). IDS patients showed no significant change in postoperative 90-day mortality over time.

### 3.3. Perioperative Outcomes and Trends

**Figure 2:**
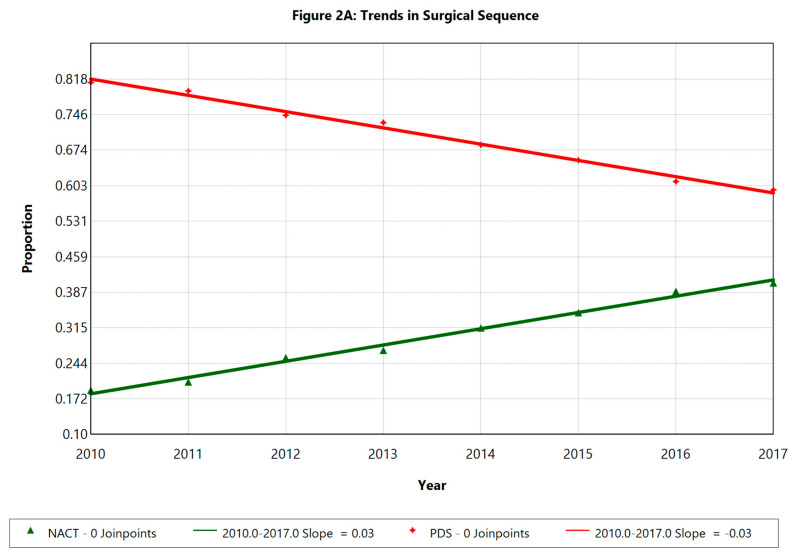

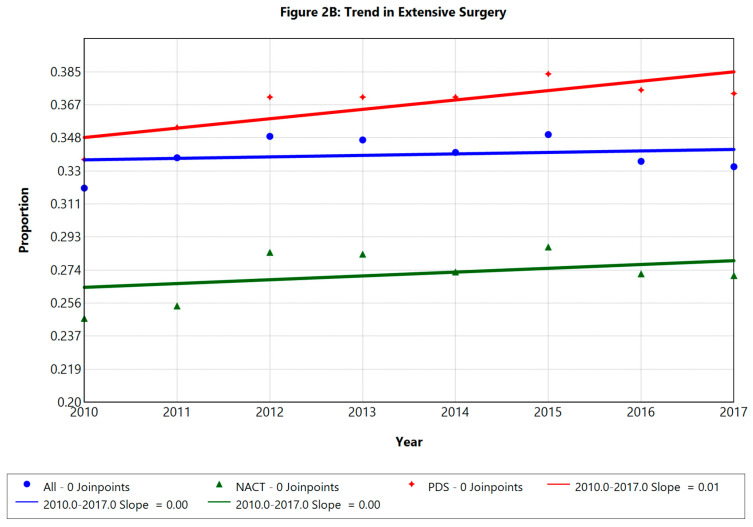
(**A**) Operative trends over time; (**B**) extensive surgery rate over time. APC = annual percentage change; NACT = neo-adjuvant chemotherapy with interval debulking surgery; PDS = primary debulking surgery.

Table 1 and Figure 2 describe the perioperative outcomes. Patients undergoing NACT with IDS had a lower 30-day hospital readmission rate (6.2% vs. 3.1%, *p* < 0.001), a shorter hospital stay (4 vs. 6 days, *p* < 0.001) and were less likely to undergo extensive surgery (27.4% vs. 36.7%, *p* < 0.001). Despite less extensive surgical intervention, patients undergoing NACT with IDS were more likely to achieve complete gross resection, or R0 (42% vs. 38.6%, *p* < 0.001).

Over the study period, the rate of IDS increased from 18.9% to 40.6% (annual change: 11.8%, *p* < 0.05). The R0 resection rate for the entire cohort rose from 34.8 to 41% (annual change: 2.65%, *p* < 0.01). This improvement in R0 resection rates was driven by improvements in PDS patients (annual improvement: 2.83%, *p* < 0.01), with no significant change in the IDS group. There was no significant trend in the rate of extensive surgery across either group over time.

## 4. Discussion

PDS was associated with significantly improved survival of 15.2 months in this large NCDB study examining treatment of women with advanced EOC (54 vs. 38.8 months). Prior retrospective studies have also shown improved survival with PDS compared to NACT with IDS [2,12]. However, this contrasts with three clinical trials showing noninferior survival of PDS versus NACT with IDS in advanced EOC. The Vergote et al. trial started enrolling patients in 1998 and reported median OS data of 29 months in the PDS group vs. 30 months in the NACT group [7]. The CHORUS trial started accruing patients in 2003 and reported median OS of 23 vs. 24 months (PDS vs. IDS) [8]. These trials hypothesized a lower-than-expected median OS compared to retrospective data due to a variety of factors, possibly due to increased age, a higher proportion of poorly differentiated tumors, worse Eastern Cooperative Oncology Group (ECOG) status, and larger tumor size [7,8].

The SCORPION trial started accruing patients in 2011 and provided longer median OS data of 41 months in the PDS group vs. 43 months in the NACT group [9]. This longer median OS is possibly attributable to use of emerging treatment options such as bevacizumab and poly ADP ribose polymerase (PARP) inhibitors that were being incorporated into the standard of care during the time this trial was published [9]. Since our retrospective data set spanned the years 2010–2017, it is most comparable in time and OS outcomes with the SCORPION trial data. Given the discrepancy between retrospective data and the prospective clinical trial data with respect to OS, these studies must be interpreted based on their level of evidence and probability of bias. Randomized controlled trials provide the highest level of scientific evidence, due to their ability to reduce bias and be less prone to systematic errors [16].

Regarding surgical trends, our large retrospective cohort study showed an increase in the use of NACT with IDS 11.8% each year from 18.9% to 40.6% and corresponding decrease in PDS over the eight-year period from 2010 to 2017. These trends are similar to previously reported NCDB data [14,17]. Ninety-day postoperative mortality decreased due to a reduction in mortality among the PDS patient cohort, suggesting improved case selection favoring the NACT with IDS approach in certain subgroups of patients.

Several experts have proposed criteria using clinical and/or imaging factors to triage newly diagnosed advanced ovarian cancer patients to PDS vs. NACT with IDS to improve oncologic outcomes [18,19,20,21]. The Mayo triage algorithm uses clinical factors of age > 80, albumin <3.5 g/dL or age 75–79 with performance status >1 or stage IV disease or high likelihood of complex surgery to determine risk of surgical morbidity and recommend either the PDS or NACT with IDS approach [18]. The MSK resectability algorithm uses a combination of three patient factors (age > 60, CA-125 > 500 u/mL, and ASA score 3–4) and seven disease factors detected on pre-operative CT scan to predict suboptimal cytoreduction [20,21]. There is no universally agreed upon criteria to triage patients to PDS or NACT with IDS. Currently, each patient is individually assessed in the context of their disease burden on imaging, clinical factors, surgeon factors, and institutional capabilities to determine the best approach. Oncologic outcomes have improved in advanced ovarian cancer over time likely due to a combined effect of these algorithms, increasing surgeon experience over time and the addition of maintenance treatments such as PARP inhibitors and bevacizumab.

In our cohort, NACT with IDS was associated with a statistically lower rate of extensive surgery compared to PDS each year. However, over time the surgical complexity remained unchanged amongst the PDS and IDS groups. This data is consistent with previous findings showing the use of NACT was associated with a reduction in operative time, fewer operative complications, and fewer organ resections [10]. However, this data contrasts with a previously reported study by Horner et al., who showed an increase in the overall complexity of ovarian cancer surgery in their analysis of the NCDB during the earlier time period of 2004–2015 [14].

The rate of complete gross resection (R0) in this cohort was 38.6% in the PDS group and 42% in the NACT group. Our R0 rate was similar to compared to supplemental trial data published by Vergote, CHORUS, JCOG 0602, and SCORPION where R0 rates were 12–47.6% in the PDS group and 39–77% in the NACT with IDS group [7,8,9,10]. Over time, the PDS R0 cytoreduction rate in our study improved from 34.8% in 2010 to 41% in 2017. The NACT R0 cytoreduction rate in our study was 42% and remained unchanged over time. This is suggestive of improved case selection and triage of patients from PDS to NACT when not a candidate for optimal cytoreduction upfront. While R0 rates are listed in the NCDB, the clinically meaningful metric of optimal tumor debulking to R < 1 cm of disease is not available. This limits further analysis and comparisons to clinical trials on the resectability of this patient population.

Drawbacks to our study include the limited availability of certain clinical and treatment factors within the database, and limitations of the generalizability of the data given the population of patients captured in the NCDB. While the NCDB captures a large amount of data, there could be unmeasured confounders such as tumor biology or genetics to explain the survival differences amongst the PDS and NACT with IDS groups found in this study. In addition, the NCDB does not capture data on performance status, which may be a contributor to the lower overall survival rates seen in the NACT with IDS population in this study. The NCDB does not capture detail about which patients received adjuvant systemic PARP inhibitors or bevacizumab, which have improved PFS in certain population subgroups, such as those with germline BRCA mutations or HRD-positive tumors [22,23,24,25]. Another potential confounder is the use of heated intraperitoneal chemotherapy (HIPEC) during debulking surgery, as this has been shown to improve PFS and OS when added to IDS [26].

There are also inherent limitations within the NCDB data based on the patients that are captured in the registry. The database only includes about 70% of patients in the US who receive ovarian cancer treatment within six months of diagnosis. NCDB only covers data from approximately 30% of all US hospitals, because it requires accreditation by the Commission on Cancer to be included in the data repository. Known populations that are underrepresented in the NCDB include American Indians, Alaska Natives, Hispanics, patients from western states and rural areas, and patients over age 85 [27]. Additionally, this data has limited generalizability to populations outside of the US, where other factors may contribute more significantly to the differences in patient outcomes.

## 5. Conclusions

Our study shows the trends in the surgical and systemic management of advanced ovarian cancer following three landmark clinical trials showing non-inferiority of NACT with IDS to PDS and adjuvant chemotherapy. The use of NACT with IDS increased year-over-year, while the 90-day mortality decreased and overall survival increased. The reduction in 90-day mortality was primarily driven by the PDS group, suggesting improvement in triage and case selection. Similarly, the R0 resection rate improved for the PDS group with no change in the IDS group, suggesting improved case selection. Triaging those patients with extensive disease burden at diagnosis to NACT and IDS may improve their chance of R0 resection and therefore the optimal surgical outcomes when debulking is attempted.

## Figures and Tables

**Table 1:** Demographics and peri-operative outcomes.

		NACT (n = 10,460)	PDS (n = 24,522)	*p*-Value
Age at Diagnosis		65 (57–72)	61 (53–69)	<0.001
Race	White	8955 (85.6%)	21,307 (86.9%)	0.015
	Black	871 (8.3%)	1811 (7.4%)	
	Asian/Pacific Islander	396 (3.8%)	906 (3.7%)	
	Other	150 (1.4%)	322 (1.3%)	
	Unknown	88 (0.8%)	176 (0.7%)	
Ethnicity	Not Hispanic	9607 (91.8%)	22,407 (91.4%)	0.022
	Hispanic/Presumed Hispanic	642 (6.1%)	1500 (6.1%)	
	Unknown	211 (2%)	615 (2.5%)	
Insurance Status	No Insurance	284 (2.7%)	895 (3.6%)	<0.001
	Private Insurance	4211 (40.3%)	12,322 (50.2%)	
	Medicaid/Medicare/Other Public	5764 (55.1%)	10,901 (44.5%)	
	Unknown	201 (1.9%)	404 (1.6%)	
Charleson Deyo Score ≥ 1		2096 (20%)	5214 (21.3%)	0.01
Diagnosis Year	2010	778 (7.4%)	3335 (13.6%)	<0.001
	2011	862 (8.2%)	3328 (13.6%)	
	2012	1081 (10.3%)	3159 (12.9%)	
	2013	1223 (11.7%)	3313 (13.5%)	
	2014	1424 (13.6%)	3082 (12.6%)	
	2015	1556 (14.9%)	2937 (12%)	
	2016	1802 (17.2%)	2831 (11.5%)	
	2017	1734 (16.6%)	2537 (10.3%)	
Length of Stay		4 (3–7)	6 (4–8)	<0.001
No Gross Residual		4388 (42%)	9456 (38.6%)	<0.001
Extensive Surgery		2811 (27.4%)	8811 (36.7%)	<0.001
Laparoscopic Evaluation		204 (2%)	891 (3.6%)	<0.001
Stage	3C	5099 (48.7%)	18,289 (74.6%)	<0.001
	4	5361 (51.3%)	6233 (25.4%)	
Readmission at 30 days		323 (3.1%)	1519 (6.2%)	<0.001
90-Day Mortality		250 (2.4%)	409 (1.7%)	<0.001
Median Overall Survival (months)		38.8 (38.0–39.6)	54.0 (53.0–54.9)	<0.001

## Data Availability

The data presented in this study are openly available in the national cancer database (NCDB).

## Supplementary Materials

The following supporting information can be downloaded at: https://www.mdpi.com/article/10.3390/cancers17172884/s1, Figure S1: Trends in Surgical Outcomes and Overall Survival among Women Undergoing Debulking Surgery for Advanced Ovarian Cancer in the U.S: Analysis of the National Cancer Database.

## Abbreviations

The following abbreviations are used in this manuscript:

OS	Overall survival
NACT	Neoadjuvant chemotherapy
PDS	Primary debulking surgery
IDS	Interval debulking surgery
EOC	Epithelial ovarian cancer
NCDB	National Cancer Database
SEER	Surveillance, Epidemiology, and End Results
