# Supplementary material for: Trends in Surgical Outcomes and Overall Survival Among Women Undergoing Debulking Surgery for Advanced Ovarian Cancer in the U.S: Analysis of the National Cancer Database
Source: Cancers (Basel). 2025 Sep 2;17(17):2884. doi: 10.3390/cancers17172884 (PMC12427705; doi:10.3390/cancers17172884)
Supplement: Supplementary file 1 [file cancers-17-02884-s001.zip › cancers-3802257-supplementary.pdf]

Supplementary Data Figure S1:

Trends in Surgical Outcomes and Overall Survival among Women Undergoing Debulking Surgery for Advanced Ovarian Cancer in the U.S: Analysis of the National Cancer Database

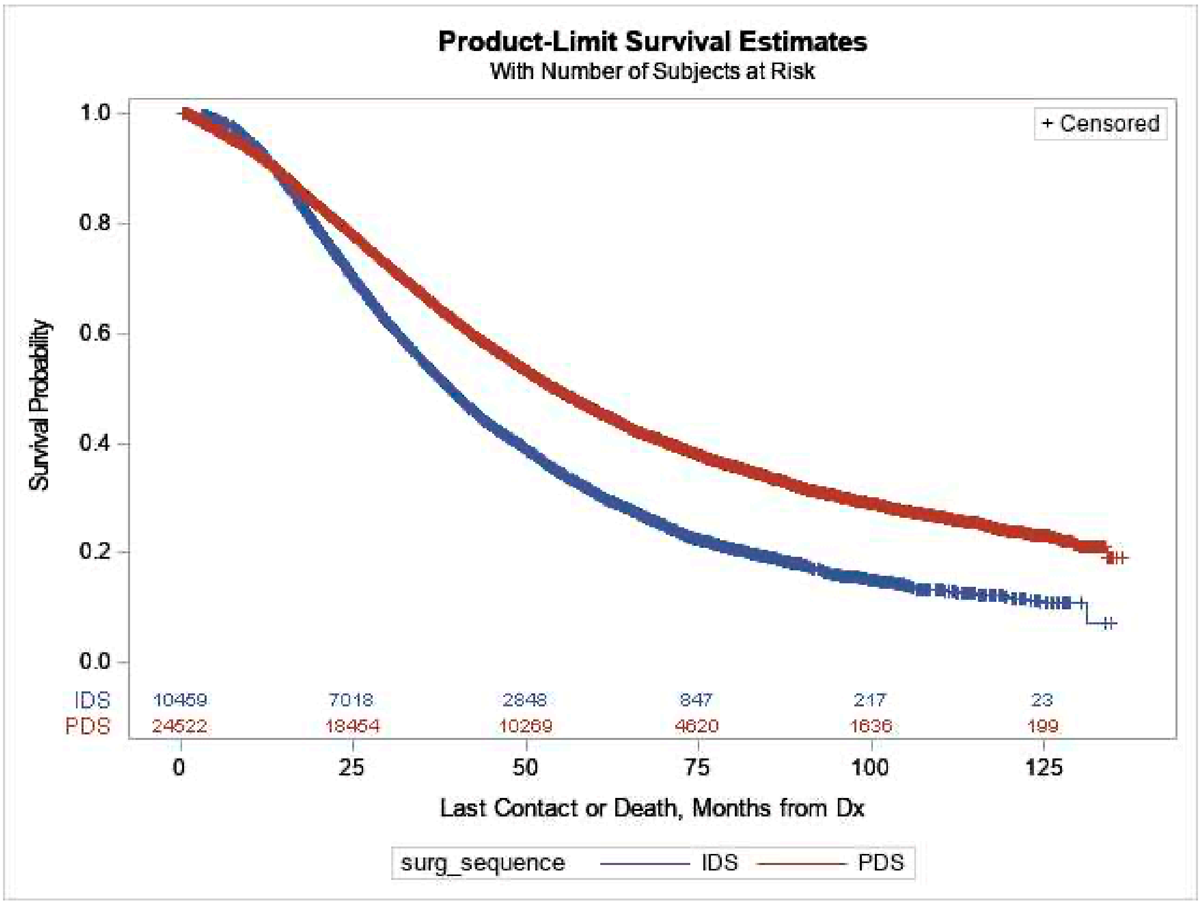

Supplementary data: Kaplan-Meier overall survival curve. IDS = interval debulking surgery, PDS = primary debulking surgery.
